# Scalp acupuncture regulates functional connectivity of cerebral hemispheres in patients with hemiplegia after stroke

**DOI:** 10.3389/fneur.2023.1083066

**Published:** 2023-05-25

**Authors:** Dan Lin, Jinyang Gao, Mengxin Lu, Xiao Han, Zhongjian Tan, Yihuai Zou, Fangyuan Cui

**Affiliations:** ^1^Department of Neurology, Dongzhimen Hospital, Beijing University of Chinese Medicine, Beijing, China; ^2^Department of Radiology, Dongzhimen Hospital, Beijing University of Chinese Medicine, Beijing, China

**Keywords:** stroke, resting-state functional magnetic resonance imaging, hemiplegia, scalp acupuncture, functional connectivity

## Abstract

**Background:**

Stroke is a common cause of acquired disability on a global scale. Patients with motor dysfunction after a stroke have a reduced quality of life and suffer from an economic burden. Scalp acupuncture has been proven to be an effective treatment for motor recovery after a stroke. However, the neural mechanism of scalp acupuncture for motor function recovery remains to be researched. This study aimed to investigate functional connectivity (FC) changes in region of interest (ROI) and other brain regions to interpret the neural mechanism of scalp acupuncture.

**Methods:**

Twenty-one patients were included and randomly divided into patient control (PCs) and scalp acupuncture (SAs) groups with left hemiplegia due to ischemic stroke, and we also selected 20 matched healthy controls (HCs). The PCs were treated with conventional Western medicine, while the SAs were treated with scalp acupuncture (acupuncture at the right anterior oblique line of vertex temporal). All subjects received whole-brain resting-state functional magnetic resonance imaging (rs-fMRI) scan before treatment, and the patients received a second scan after 14 days of treatment. We use the National Institutes of Health Stroke Scale (NIHSS) scores and the analyses of resting-state functional connectivity (RSFC) as the observational indicators.

**Results:**

The contralateral and ipsilateral cortex of hemiplegic patients with cerebral infarction were associated with an abnormal increase and decrease in basal internode function. An abnormal increase in functional connectivity mainly exists in the ipsilateral hemisphere between the cortex and basal ganglia and reduces the abnormal functional connectivity in the cortex and contralateral basal ganglia. Increased RSFC was observed in the bilateral BA6 area and bilateral basal ganglia and the connectivity between bilateral basal ganglia nuclei improved. However, the RSFC of the conventional treatment group only improved in the unilateral basal ganglia and contralateral BA6 area. The RSFC in the left middle frontal gyrus, superior temporal gyrus, precuneus, and other healthy brain regions were enhanced in SAs after treatment.

**Conclusion:**

The changes in functional connectivity between the cerebral cortex and basal ganglia in patients with cerebral infarction showed a weakening of the bilateral hemispheres and the enhancement of the connections between the hemispheres. Scalp acupuncture has the function of bidirectional regulation, which makes the unbalanced abnormal brain function state restore balance.

## Background

With the improving medical conditions, stroke remains a leading cause of death and long-term disability worldwide. Epidemiologic evidence shows that the average global lifetime risk of stroke has increased from 22.8 to 24.9% over the past 20 years; in the United States, the prevalence of stroke in adults is 3.0% (median). On average, someone suffers a stroke nearly every 40 s, and rates increase with age in both men and women (1). In China, stroke has become the leading cause of death in 2019 (2, 3). Hemiplegia is one of the most common sequelae after stroke, which directly affects the quality of work and life of patients and imposes a heavy economic burden on society and families (4).

Motor impairment is the most common symptom of stroke. In recent years, motor impairment is found to be relevant to abnormal functional connectivity in several functional magnetic resonance imaging (fMRI) studies (5–7). The correlations of the interhemispheric FC changes during motor function injury and rehabilitation have been observed widely, especially between the brain regions directly related to the motor network. Many previous studies showed (8, 9) that interhemispheric functional connectivity in the motor network is a potential neurobiological marker for recovery after stroke rehabilitation. However, the recovery of motor function and the reconstruction of the movement patterns are also relative to a wider range of functions, such as perception, memory, learning, executive functioning, and emotion management (10–12).

Spontaneous behavioral recovery usually occurs within weeks to months after stroke, which is often incomplete and affected by age, previous comorbidities, and the size and location of the injury (13). A large number of studies have demonstrated that appropriate rehabilitation therapy could promote the recovery of motor function, thus enhancing the functional recovery of damaged areas by promoting neuroplastic changes. The mechanisms of these therapies are similar to the mechanisms observed in spontaneous recovery (14). Some researches show that acupuncture may be possible to promote motor recovery by coordinating functional activities of more extensive brain regions, which initially were involved in executive function processes such as planning, initiation, and attention (15, 16). At present, scalp acupuncture is widely used in the clinical treatment of stroke and rehabilitation of stroke (17). According to the traditional Chinese medical theory, the head is where the IQ and the meridian merge. Scalp acupuncture can improve the excitability of the central nervous system, effectively improve cerebral hemodynamics and brain tissue metabolism, and then promote the recovery of motor function of patients. In this study, we evaluated the role of Scalp acupuncture treatment in patients with hemiplegia after stroke and analyzed the changes in RSFC. This study may help to evaluate the mechanism of scalp acupuncture treatment in patients with hemiplegia.

## Materials and methods

### Participants

This study was approved by Dongzhimen Hospital Affiliated with the Beijing University of Chinese Medicine Institutional Review Boards (approval number: ECSL-BDY-2014-16).

Twenty-one patients with left hemiplegia due to ischemic stroke were recruited from the Dongzhimen Hospital (Beijing, China) between February 2014 and December 2017. In addition, 20 healthy subjects were recruited as a control group with matched age, sex, and education levels. Patients were randomly classified by using the random number table method.

Inclusion criteria: (a) patients had a clinically diagnosed by computed tomography or MRI first episode of ischemic stroke involving unilateral partial anterior circulation infarction; (b) patients were between 40 and 75 years old and right-handed; (c) patients' course was between 2 weeks and 6 months after the onset of stroke; (d) patients had the stable physical condition without disorders in consciousness or speech and can understand consenting and study-related instructions; (e) patients had signed the informed consent form. Exclusion criteria: (a) patients with a course of disease longer than 6 months or in the acute phase of disease progression; (b) patients with sensory aphasia, severe dementia, psychosis, and so on, which may affect the experiment; (c) patients with a cardiac pacemaker, stent, bypass surgery, or otherwise, which were not available for magnetic resonance examination.

### Interventional method

Both groups were given conventional Western medicine treatment, such as blood pressure-lowering treatment, lipid-lowering and stabilizing agents, and anti-platelet.

Acupuncture was applied at the anterior oblique line of vertex temporal (MS6) according to the International Standardization Scheme for Scalp Acupuncture Points (18). First, we sterilize the skin of patients and our hands, and then, the stainless steel needles (0.25 × 40 mm) were inserted at angles of almost 15° with respect to the epicranial aponeurosis. When the needle goes into the scalp to a certain depth, the needles were twisted at a frequency of 200 turns per min, causing a deqi sensation. Twist the needles every 10 min for 30 min. Once a day for 5 consecutive days as a course of treatment.

### Study procedure

**Figure 1:**
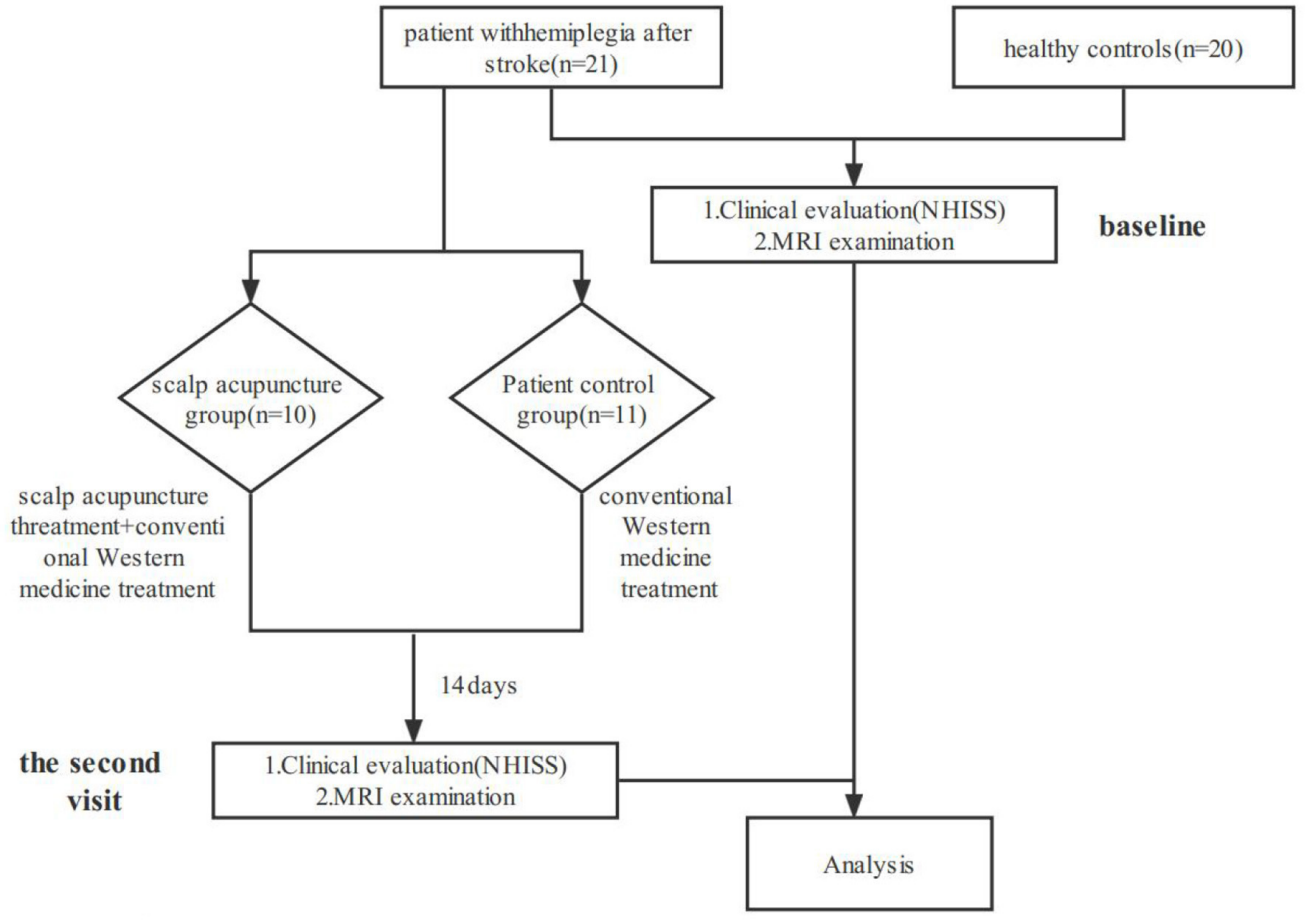
Flow chart of the current study.

All subjects underwent the National Institute of Health stroke scale (NIHSS) evaluations and their first rs-fMRI examination at baseline; patients with hemiplegia underwent the second NIHSS evaluation and rs-fMRI examination after scalp acupuncture treatment. The flow chart of this study is shown in Figure 1.

### Image data acquisition

MRI data were acquired using a 3.0 Tesla Siemens scanner (MAGNETOM Verio Siemens Medical Systems, Erlangen, Germany) with a 32-channel head coil at the Dongzhimen Hospital, Beijing, China. For rs-fMRI scans, participants were instructed to keep their eyes closed and stay awake without performing any cognitive tasks. The imaging parameters of the EPI sequence were as follows: repetition time (TR) = 2,000 ms, field of view (FOV) = 250 × 250 mm^2^, echo time (TE) = 30ms, slice number = 31, thickness = 3.5 mm, flip angle = 90°, and matrix size = 64 × 64. The single-acquisition time was 620 s. High-resolution structural images were acquired through a magnetization-prepared rapid acquisition with gradient-echo (MPRAGE) sequence with the following parameters: TR/TE = 2,700/2.97 ms, FOV = 250 × 250 mm^2^, matrix size = 256 × 256, flip angle = 7°, slice number = 176, and slice thickness = 1 mm. The single-acquisition time was 377 s.

### FMRI data analysis

The preprocessing of fMRI data was performed with SPM12 (https://www.fil.ion.ucl.ac.uk/spm/software/spm12/) in MATLAB 8.2 (https://ww2.mathworks.cn/products/matlab.html). First, we preprocessed the FMRI data, mainly including the following steps: (a) the data of the first 10 time points were deleted to ensure the stability of MRI data; (b) slice time correction and motion correction (over 1 mm or 1° were excluded); (c) spatial normalization; (d) smoothing of the data with an 8-mm full width at half maximum (FWHM) kernel; (e) detrend and filtering, band-pass filtering was performed with a frequency window of 0.008–0.09 Hz; and (f) in addition to signals of interest, confounding factors were eliminated by segmentation of white matter and cerebrospinal fluid (CSF) regions.

Seven regions of the subcortex that were widely used in previous studies were examined (19–22). These seven seeds, including the caudate nucleus, putamen, nucleus accumbens, globus pallidus, subthalamic nucleus, substantia nigra, and thalamus, were extracted using WFU-Pick Atlas software (https://www.nitrc.org/projects/wfu_pickatlas).

Functional connectivity analysis was carried out using a seed-based approach in the CONN16b (https://www.nitrc.org/projects/conn/). The first-order correlation map was generated by extracting the remaining BOLD time process from each striatum seed region and calculating its Pearson correlation coefficient with the whole brain voxel time process. The correlation coefficients were converted to “Z” values by Fisher conversion, and their normality was improved to enable them to be used in improved second-order general linear model analysis. In addition, the differences in FC between ROI points and the whole brain between patients and HCs were compared by the two-samples *t*-test in the secondary group analysis. Voxel threshold *p* < 0.005 (uncorrected) and cluster level *p* < 0.05 (FDR corrected) were considered as statistical differences. SPSS18.0 statistical software package was used for clinical data analysis; the chi-square test was used for counting data, *t*-test was used for measurement data consistent with normal distribution and Mann–Whitney test was used for non-parametric test if not consistent with normal distribution. *P* < 0.05 was considered statistically significant.

## Results

### Demographic and clinical data

The demographic and clinical characteristics of the participants are summarized in Table 1. There were no significant differences between the patients and HCs in terms of age (*p* = 0.079) and gender (*p* = 0.444), and between the scalp acupuncture (SAs) group and patient control (PCs) group in terms of gender (*p* = 0.696). There were significant differences between SAs and PCs in terms of age (*p* = 0.003). At the same time, no significant differences (*p* = 0.410) were found between the SAs and PCs in the NIHSS scores before the first scan, while significant differences were found (*p* = 0.027) after 14-day treatment (Table 1).

**Table 1:** Demographics and clinical characteristics of patients with hemiplegia after stroke and healthy controls (HCs).

	**HCs (*n* = 20)**	**SAs (*n* = 11)**	**PCs (*n* = 10)**
Age	57.4 ± 8.39	68.4 ± 4.5	57.1 ± 9.8
Sex (female/male)	7/13	2/8	3/8
NIHSS score	NA	5.0 ± 2.75	5.9 ± 2.62

NA, not available. The data were expressed as (Mean ± SD).

### Functional connectivity results

#### Decrease RSFC between contralateral hemispheres

##### Caudate nucleus

**Figure 2:**
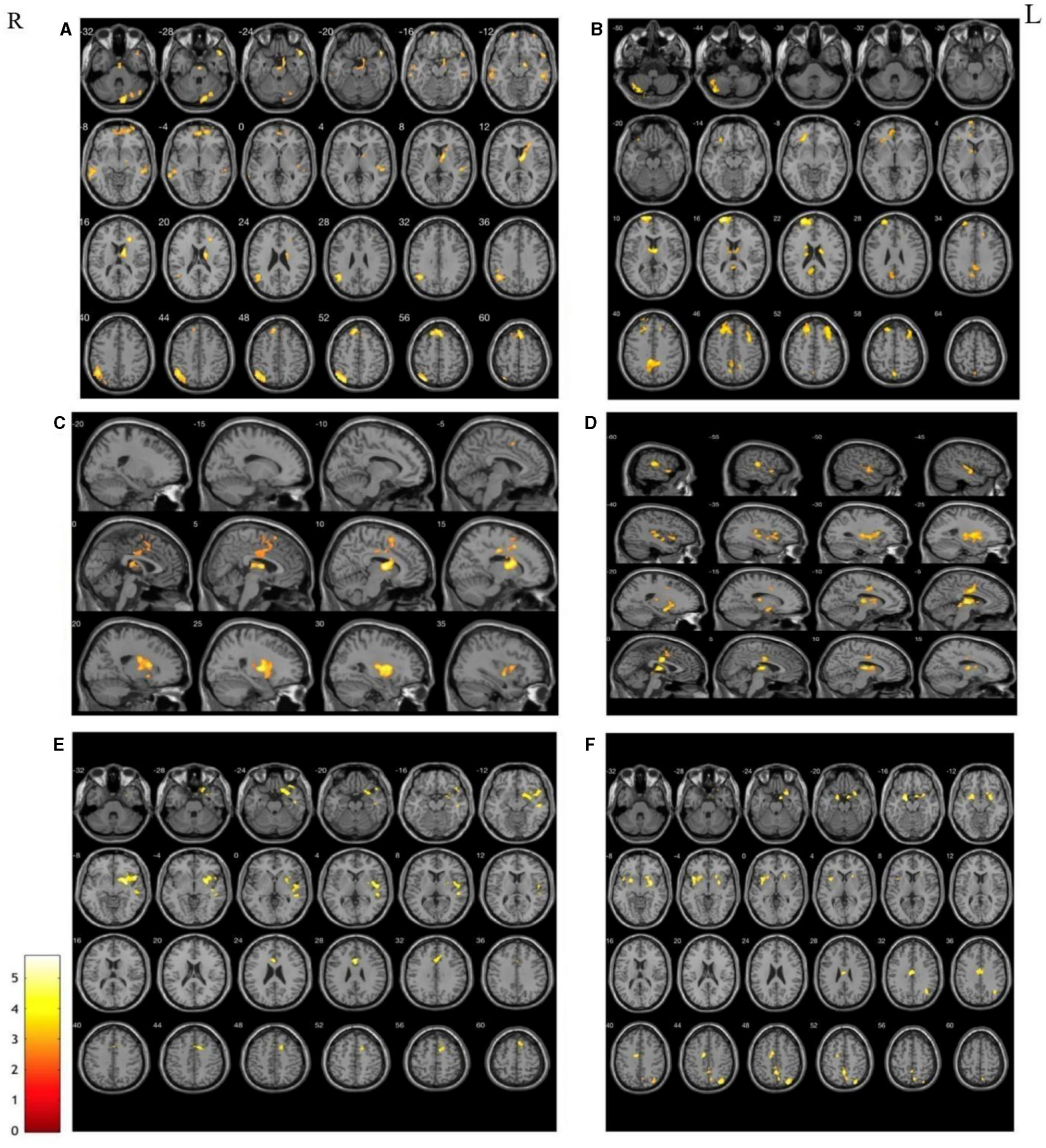
The areas with the significantly reduced between HCs and patients in terms of the changes in functional connectivity before treatment. Decreased RSFC were found in both left and right seeds, including caudate nucleus **(A, B)**, putamen **(C, D)** and thalamus **(E, F)**. Interestingly, the weakened connectivity areas mainly located on the contralateral cerebral hemisphere of the seed point.

Significantly decreased RSFC was found between the left caudate and right posterior cerebellar lobe, right caudate nucleus, right temporal lobe, right medial frontal gyrus, left parietal lobe, left superior frontal gyrus, and left temporal lobe in stroke patients compared to the HCs. Decreased RSFC was observed between the right caudate and left middle frontal gyrus, left thalamus, left frontal lobe, left posterior cerebellum, left cingulate gyrus, and right middle frontal gyrus (Figures 2A, B).

##### Putamen

Compared to HCs, decreased RSFC was also observed between the putamen and contralateral areas in stroke patients with left hemiplegia. Interestingly, both sides showed weakened RSFC between the putamen and contralateral thalamus and cingulate gyrus. In other words, there was a significantly reduced RSFC of the left putamen with the right thalamus and right cingulate gyrus and of the right putamen with the left thalamus and left cingulate gyrus in patients with left hemiparesis (Figures 2C, D).

##### Thalamus

Decreased RSFC of the left thalamus and the right frontal lobe, right temporal lobe, and right cingulate gyrus were observed in patients with stroke. Furthermore, there was decreased RSFC between the right thalamus and the left parahippocampal gyrus, left cingulate gyrus, right parietal lobe, and right anterior cuneal lobe (Figures 2E, F).

#### Increased RSFC in the ipsilateral hemisphere

**Figure 3:**
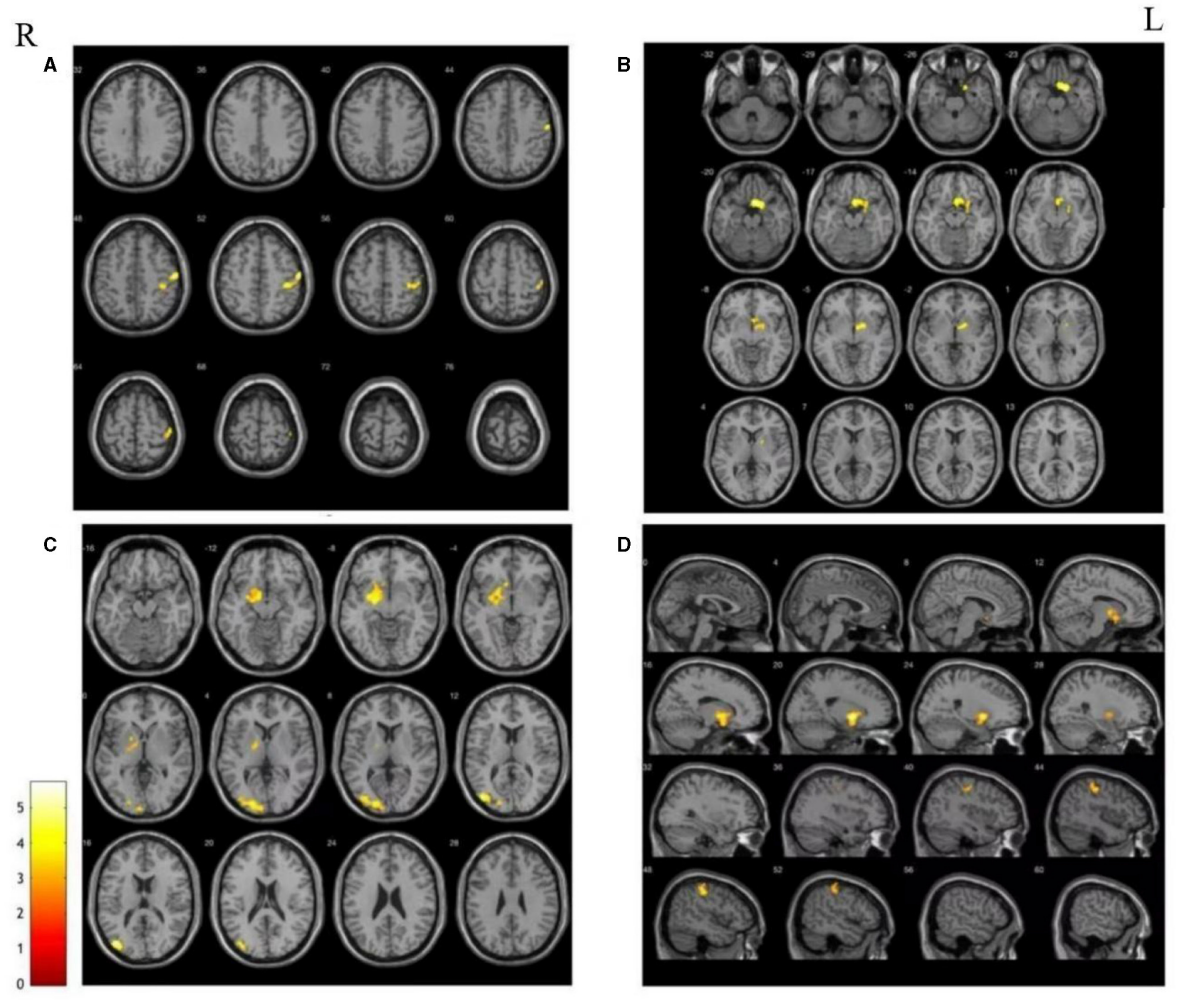
The areas with the significantly increase between HCs and patients in terms of the changes in functional connectivity before treatment. **(A)** Increased RSFC between right putamen and right parietal; **(B)** Increased RSFC between right nucleus accumbens and the right pallidum; **(C)** increased RSFC between the left globus pallidus and the left lateral globus pallidus and the middle occipital gyrus; **(D)** increased RSFC of the right globus pallidus with the right putamen and right parietal lobe.

Contrary to the weakened RSFC, significantly increased RSFC was found in the patients located within the same side of the seed points compared with HCs. There was increased RSFC between the right putamen and the right parietal lobe; increased RSFC between the right nucleus accumbens and the right pallidum; increased RSFC between the left globus pallidus and the left lateral globus pallidus and the middle occipital gyrus; and increased RSFC of the right globus pallidus with the right putamen and right parietal lobe (Figure 3).

#### Changes of RSFC in patients after treatment

##### Patients treated with acupuncture

Having received a 14-day treatment with scalp acupuncture, patients in the acupuncture group showed both increased and decreased RSFC of different seeds.

There were increased RSFC between the left caudate nucleus and the left median frontal gyrus, increased RSFC between the right globus pallidus and the left frontal gyrus, decreased RSFC between the right thalamus and the left temporal lobe, and decreased RSFC between the left caudate nucleus and the left superior temporal gyrus and left marginal lobe (Table 2).

**Table 2:** Brain regions with increased and reduced functional connectivity after treatment in the SAs.

**Seed**		**Region**	**Hem/BA**	***T***	***Z***	**Voxels**	**Coordinate MNI (mm)**		
							***X***	***Y***	***Z***
Increase	Cau(L)	Middle frontal gyrus	R/BA9,6	4.34	3.59	566	50	24	34
	Glo(R)	Superior frontal gyrus	L/BA6,10	4.99	3.90	375	−26	27	−2
Reduce	Tha(L)	Temporal lobe	L/BA39	6.12	4.44	510	−44	−58	22
	Cau(L)	Superior temporal gyrus	L/BA39,40	5.95	4.37	489	−42	−58	28
		Limbic lobe	L/BA31	4.47	3.62	379	−4	−52	32

R, right; L, left; BA, Brodmann area; Cau, caudate nucleus; Tha, thalamus; Glo, globus pallidus.

##### Patients without acupuncture

Compared with HCs, increased RSFC of the right globus pallidus with the right suboccipital gyrus, the left superior frontal gyrus, and decreased RSFC of the left globus pallidus with the right superior frontal gyrus were observed in patients after 14-day non-acupuncture treatment (Table 3).

**Table 3:** Brain regions with increased and reduced functional connectivity after treatment in the PCs.

**Seed**		**Region**	**Hem/BA**	***T***	***Z***	**Voxels**	**Coordinate MNI (mm)**		
							***x***	***y***	***z***
Increase	Glo(R)	Inferior occipital gyrus	R/BA17,18	5.96	4.37	482	30	−90	−18
		Superior frontal gyrus	L/BA6	4.66	3.73	420	−22	58	−4
Reduce	Glo(L)	Superior frontal gyrus	R/BA6	4.88	3.85	343	18	−8	72

R, right; L, left; BA, Brodmann area; Glo, globus pallidus.

#### RSFC between SAs and PCs

**Figure 4:**
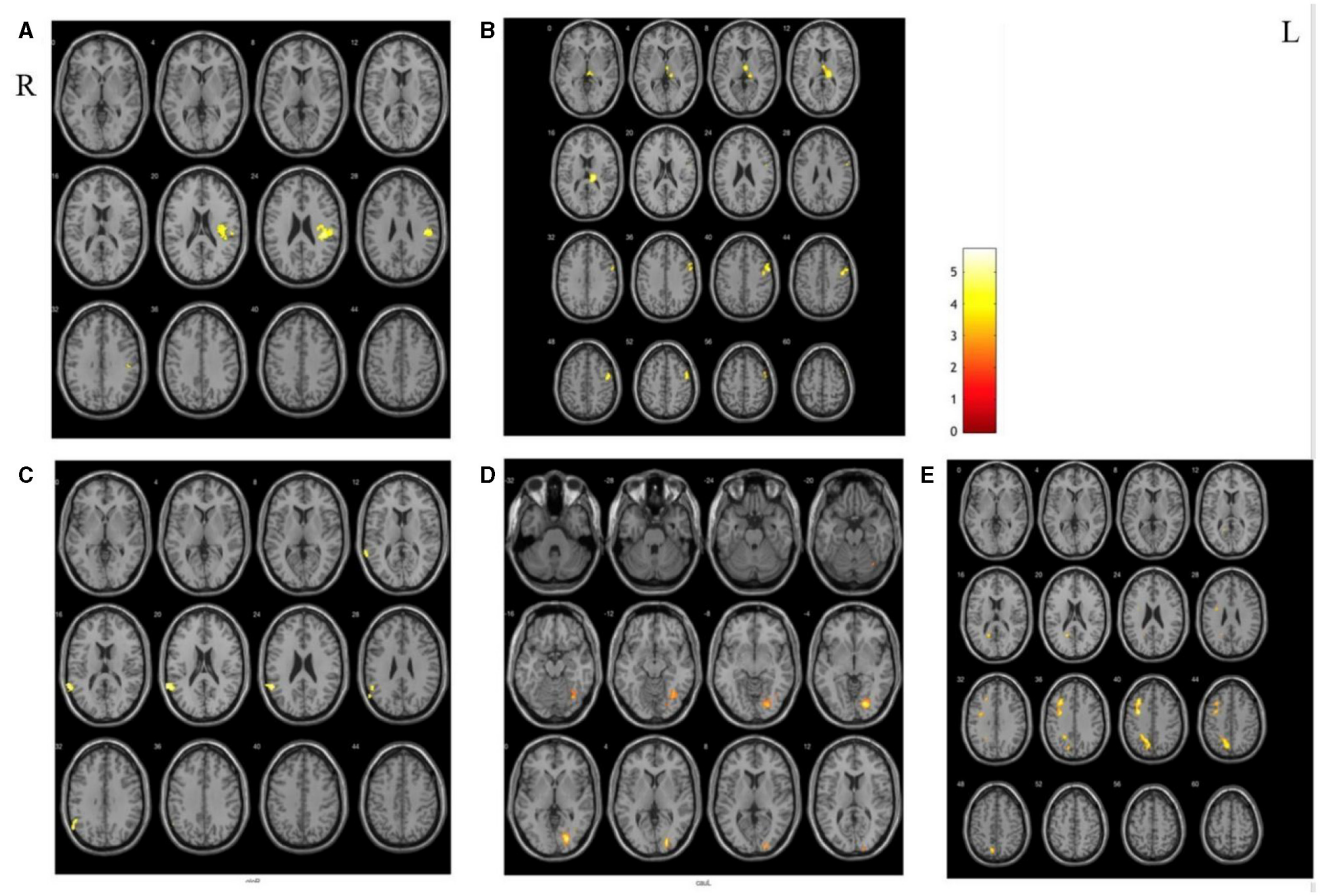
The different areas of functional connectivity between SAs and PCs after treatment. **(A, B)** Increased RSFC between SAs and PCs; **(C–E)** deceased RSFC between SAs and PCs.

Compared with the PCs, there were significant differences in extensive brain regions of RSFC in SAs after treatment, and their localizations were as follows: (a) increased RSFC of the right putamen with the right posterior central gyrus and insula; (b) increased RSFC of the left thalamus with the right thalamus, the occipital thalamus, and the precentral gyrus; (c) decreased RSFC of the left globus pallidus with the left superior temporal gyrus; (d) decreased RSFC of the left caudate nucleus with the right lingual gyrus; (e) decreased RSFC of the left thalamus with the left middle frontal gyrus and precuneus (Figure 4).

## Discussion

In this study, we found that there were significant differences between SAs and PCs in the NIHSS scores after scalp acupuncture treatment. This suggested that scalp acupuncture may have a great effect on the improvement of neurological dysfunction in patients with hemiplegia, which is consistent with the results of previous studies (23). At the same time, this study provides new insights into the functional connectivity of the cerebral hemispheres after a stroke and advances our understanding of the functional reorganization of the brain after a stroke and the promotion of motor rehabilitation by scalp acupuncture. In addition to local tissue damage, changes in functional connectivity between intact structural regions that connect the site of injury could also cause motor dysfunction in patients with spotty brain injuries (24).

The functional connectivity strength of each brain voxel is closely related to behavioral deficits and is an important predictor of post-stroke recovery. Compared with the HCs, the FC between basal ganglia nuclei with the contralateral brain regions was significantly decreased in patients with hemiplegia after stroke, especially in similar areas of the two brain hemispheres, which is consistent with previous research (25, 26). Damage of the unilateral brain in patients can affect the exchanges and cooperation between the hemispheres of the brain. At the same time, we also found that the FC was increased between the sensory and visual-related brain areas in the ipsilateral hemisphere on both affected and healthy sides in hemiplegia patients, which suggests that the ability of patients to receive and process the sensory information of the hemiplegic side and observe the external environment was enhanced after cerebral infarction. In this study, patients were all in the convalescence period, which had certain functional recovery and compensation. The enhanced FC in the ipsilateral hemisphere of the brain was also consistent with the brain functional state of functional injury and repair in the period of recovery. In summary, the regularity changes of FC in the brain revealed the abnormal motor network after the stroke and also reflected the functional reorganization during recovery.

In the present study, the RSFC in the contralateral auxiliary motor area was enhanced in both the lesion and the healthy side of the basal ganglia after scalp acupuncture treatment. However, the functional connectivity between the basal ganglia of the lesion and the contralateral BA6 was enhanced after conventional treatment in the patient control group. The diversity of functions involving BA6, probably the largest Brodmann's area, is not surprising. The BA6 area, including the premotor cortex and supplementary motor area, affects the realization of motor function through the regulation of the primary motor cortex and is also involved in language, memory, attention, and executive functions (27, 28). Both animal experiments and clinical trials suggest that activation of the BA6 region contributes to functional reorganization after stroke (29, 30). Another clinical trial of scalp acupuncture for acute ischemic stroke suggested that after scalp acupuncture, the voxel-mirrored homotopic connectivity value of bilateral BA6 and BA8 increased significantly, and the synchronization of functional activities between bilateral BA6 and BA8 was enhanced (15). A study of regional homogeneity between acupuncture and ischemic stroke found that both groups showed higher ReHo in BA6 (31). The above studies were similar to the results of this study, which showed that scalp acupuncture could significantly improve the FC of the cortical–subcortical motor pathway and promote the recovery of motor function in patients with cerebral infarction.

The significance of hyperexcitability ipsilateral motor responses from the unaffected hemisphere for motor recovery has been considered to be doubtful (32). Although the enhanced activation of the contralateral brain region after stroke can promote functional rehabilitation in the short term, it could also cause wrong movement patterns such as associated movement, which has adverse effects on the rehabilitation of the neurological function of the damaged hemisphere (33). The better the prognosis for rehabilitation, the more the degree of abnormal hyperactivation of the healthy hemisphere is reduced during rehabilitation and the more normalized the imbalance in bilateral brain function due to lesion injury (26, 34). In this study, some brain regions in the unaffected hemisphere showed enhanced functional connectivity, while the functional connectivity between the left basal ganglia, left temporal lobe, and left marginal lobe was weakened after scalp acupuncture treatment. In addition, compared with the patient control group, the scalp acupuncture group showed a greater degree of functional connectivity decline in the left middle frontal gyrus, superior temporal gyrus, precuneus, and other healthy brain regions. The above results suggest that scalp acupuncture is more beneficial to reduce the compensation of contralateral function. Compared with conventional treatment, scalp acupuncture can significantly reduce the patient's dependence on the compensation of contralateral motor function after stroke and is conducive to the recovery of normal motor patterns of stroke patients. Furthermore, scalp acupuncture treatment not only has a stronger promotion effect on BA6 but also significantly improves the functional connection between the non-motor related brain regions such as the postcentral gyrus and insula. As an important part of the sensorimotor network, the primary somatosensory cortex is located in the postcentral gyrus and is responsible for sensory input (35). The insula involves a wide diversity of functions observed, such as pain, temperature, touch, olfaction, taste, language, memory, and emotion (36). The rehabilitation effect of scalp acupuncture on cortical function is not only limited to the motor area, but also has a positive effect on sensory and cognitive-related functions, and improves nerve function as a whole by promoting the mutual coordination of each brain functional area.

## Limitations

There are some limitations in this study. One of the limitations is that there were fewer subjects, and the other is that the patients in this study were in the recovery period of cerebral infarction, but there may be some differences in the remodeling of neural function in different stages of the recovery period. The mechanism of change of head acupuncture treatment may also change differently. Further studies with larger sample sizes and more optimized research designs are needed to confirm our findings.

## Conclusion

Scalp acupuncture treatment has a bidirectional adjustment function and promotes rehabilitation by strengthening functional connections of the bilateral motor cortex and weakening abnormal compensatory connections.

## Data availability statement

The original contributions presented in the study are included in the article/supplementary material, further inquiries can be directed to the corresponding author.

## Ethics statement

The studies involving human participants were reviewed and approved by the Ethics Committee of the Dongzhimen Hospital Affiliated to Beijing University of Chinese Medicine Institutional Review Boards (approval number: ECSL-BDY-2014-16). The patients/participants provided their written informed consent to participate in this study. Written informed consent was obtained from the individual(s) for the publication of any potentially identifiable images or data included in this article.

## Author contributions

DL: writing—original draft preparation, validation, project administration, and supervision. JG: software, investigation, methodology, visualization, and formal analysis. ML: investigation, methodology, and writing—review and editing. XH: data curation and formal analysis. ZT: visualization, supervision, and validation. YZ: methodology, resources, writing—review and editing, and visualization. FC: conceptualization, methodology, writing—review and editing, supervision, and funding acquisition. All authors contributed to the article and approved the submitted version.
